# Efficacy of a Novel Treatment Serum in the Improvement of Photodamaged Skin

**DOI:** 10.1111/ics.12018

**Published:** 2012-12-05

**Authors:** S Sonti, E T Makino, J A Garruto, J V Gruber, S Rao, R C Mehta

**Affiliations:** * SkinMedica, Inc5909 Sea Lion Place, Suite H Carlsbad, CA, 92010, U.S.A; † Lonza Personal Care70 Tyler Place, South Plainfield, NJ, 07080, U.S.A

**Keywords:** Anti-aging, antioxidants, peptides, photodamage, topical

## Abstract

**Synopsis:**

A novel treatment serum formulated to target multiple pathways in the anti-ageing cascade was tested both *in vitro* and in clinical settings. *In vitro* testing was performed to assess the ability to stimulate key proteins and genes fundamental to the anti-ageing cascade. The antioxidant potential of the formulation was studied in a UV-irradiation clinical study. A 12-week, open-label, single-centre study was conducted to determine whether this uniquely formulated topical treatment serum could improve visible signs of facial photodamage. Clinical evaluations showed statistically significant reductions in fine wrinkles and coarse wrinkles and improvements in skin texture, tone and radiance starting at week 4 with continued improvements at weeks 8 and 12. Subject self-assessments confirmed that the beneficial effects of the treatment serum were readily observed by the users. The treatment serum was well tolerated with no treatment-related adverse events reported during the 12-week study. Use of this novel treatment serum produced significant improvements in the visible signs of facial photodamage.

**Résumé:**

Un nouveau sérum de traitement conçu pour cibler de multiples voies dans la cascade anti-âge a été testé à la fois in vitro et dans les conditions cliniques. Les tests in vitro ont été réalisés afin d'évaluer la capacité de stimuler les protéines et les gènes clés fondamentales de la cascade anti-vieillissement. Le potentiel antioxydant de la formulation a été étudié dans une étude clinique utilisant le rayonnement UV. Une étude de douze semaines, en mode ouvert, monocentrique a été menée afin de déterminer si ce sérum spécialement formulé pour le traitement topique peut améliorer les signes visibles du photo-vieillissement du visage. Des évaluations cliniques ont montré une réduction statistiquement significative des rides et des ridules secondaires et l'amélioration de la texture de la peau, du tonus et d'éclat à partir de la semaine 4 avec des améliorations continues aux semaines 8 et 12. Les autoévaluations par les sujets ont confirmé que les effets bénéfiques du sérum de traitement étaient facilement observés par les utilisateurs. Le sérum de traitement a été bien toléré avec aucun événement indésirable rapporté au cours de l'étude de 12 semaines. L'utilisation de ce nouveau sérum de traitement produit des améliorations significatives dans les signes visibles du photovieillissement du visage.

## Introduction

Skin ageing is a cumulative and multi-factorial process, which is mediated by a combination of the effects of time (intrinsic ageing) and environmental factors (extrinsic ageing). Intrinsic ageing results from an accumulation of metabolic oxidative damage to the skin microstructure whereas extrinsic ageing results from skin exposure to UV radiation and environmental pollutants 1, 2. These two skin ageing processes have common biochemical and molecular pathways, including nuclear and mitochondrial DNA damage, breakdown of matrix proteins and membrane lipid damage 2005. New insights regarding convergence of the molecular basis of chronological ageing and photoageing provide opportunities for the development of new anti-ageing therapies.

**Figure 1:**
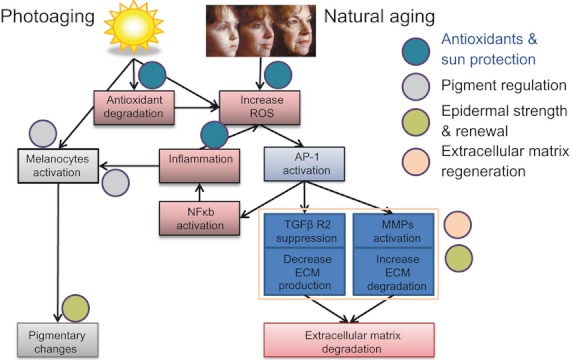
Biochemical pathways associated with extrinsic (photo-aging) and intrinsic aging. Both extrinsic and intrinsic aging share some common biochemical pathways. An ideal anti-aging topical therapy would address the following aspects: (1) Antioxidants and sun protection: Prevent UV induced sun damage, increase skin's antioxidant capacity, regenerate natural antioxidants (2) Pigment regulation: Inhibit melanin production, prevent melanocyte activation, etc (3) Epidermal strength and renewal: Increase skin barrier and hydration, increase skin cell turnover (4) Extracellular matrix regeneration: Prevent ECM degradation, induce ECM synthesis, improve cellular health and communication. (AP-1 = Activator Protein 1).

Key factors involved in ageing include the generation of reactive oxygen species (ROS), activation of transcription factors, induction of matrix metalloproteinases (MMPs), decreased synthesis of collagen and pigmentary changes (Fig. 1). Normally, the level of collagen is maintained by ensuring a balance between collagen synthesis by fibroblasts in the dermis and enzymatic degradation by MMPs 2005. Much of the progressive damage in collagen structure/function that occurs from ageing and photodamage is due to an increased expression of enzymes that degrade collagen and decreased synthesis of procollagen 2009.

The skin is constantly exposed to external and internal insults that ultimately result in damage to the extracellular matrix (ECM). Healthy, younger skin repairs this damage efficiently. However, aged skin has a reduced ability to repair the damage, resulting in up to a 20% reduction in collagen, an imbalance in the ratio type 3/type 1 collagen, loss of skin elasticity, wrinkle and fine line formation and pigmentary changes 1993.

An ideal anti-ageing treatment, as described in Fig. 1, would not only have a protective and reparative effect, but would also address overall skin health by re-establishing the balance of collagen synthesis and degradation, and maintaining the youthful nature of skin function.

With the above goal, a novel treatment serum (Biometa Essential Serum™; SkinMedica, Inc., Carlsbad, CA, U.S.A.) was formulated with a proprietary combination of scientifically proven anti-ageing ingredients in a dual-chamber airless pump. Chamber 1 of the pump contains Biometa Complex, an advanced bioscience product created through a proprietary bio-fermentation process. Chamber 2, Antioxidant Peptide Serum (APS), contains a concentrated blend of antioxidants, collagen-building peptides and depigmenting agents.

Discussed within is a methodical approach for developing an anti-ageing treatment product designed to target multiple pathways in the anti-ageing cascade. The ability of Biometa Complex (Chamber 1) to stimulate key proteins and genes fundamental to the anti-ageing cascade was first tested using *in vitro* assays. Gene and protein expression was analysed in dermal fibroblasts treated with Biometa Complex. Next, the effect of APS (Chamber 2) containing a combination of antioxidants, anti-inflammatory agents, collagen-building peptides and depigmenting agents was tested by conducting a UV-irradiation clinical study.

Lastly, the ultimate litmus test for any anti-ageing product is its effect on clinical manifestations of ageing including a loss of skin elasticity and firmness, fine lines and wrinkles, and an uneven skin tone. To explore the clinical benefits of this topical treatment serum, a twelve-week clinical study was conducted evaluating the safety and efficacy of the treatment serum when used by females with mild to severe facial photodamage.

## Patients and methods

### Methods: *In vitro* studies (Biometa Complex)

#### Gene Array Analysis

Human dermal fibroblasts were seeded into either a 96-well plate (cytotoxicity assay) or into T-25 flasks (cytokine array) and grown at 37 ± 2°C and 5 ± 1% CO_2_ using fibroblast growth media (FGM). Safe working concentration for each test material was determined using an (3-(4,5-Dimethylthiazol-2-yl)-2,5-diphenyltetrazolium bromide (MTT) assay (SkinMedica, Inc.). Upon reaching confluence, the cells were treated with the test materials for 24 h. After the 24-h treatment, total RNA was isolated using an RNAqueous Kit (Ambion) as per the manufacturer's instructions. After purification, the total RNA was prepared for array use by first amplifying the RNA using a MessageAmp aRNA Kit (Ambion), and then fluorescently labelling the aRNA with Cy3 or Cy5 using an ASAP Labeling Kit (Perkin Elmer), both as per the manufacturer's instructions. The fluorescently labelled aRNA was applied to the DNA microarray chips (Agilent Technologies, Santa Clara, CA, U.S.A.), and the chip was hybridized overnight and washed as per the manufacturer's recommended protocol. After washing, the microarrays were scanned with an Axon GenePix 4100A Scanner and analysed with GenePix Pro software. Fluorescence intensities for the microarrays were subjected to global normalization. The total fluorescent signal for both dyes was normalized with a correction factor that would make the ratio of total intensities for both dyes equal to one. For this study, a Cy3/Cy5 (untreated/treated) fluorescence intensity ratio greater than 1.3 or less than 0.7 (this relates to a change in gene expression of at least 30%) was used as the cut-off for up- and downregulated genes, respectively. In addition, the fluorescence intensity of the gene marker had to be greater than the background intensity.

#### Protein Array Analysis

Human dermal fibroblasts were seeded into either a 96-well plate (cytotoxicity assay) or into T-25 flasks (cytokine array) and grown at 37 ± 2°C and 5 ± 1% CO_2_ using FGM. Safe working concentration for each test material was determined using an MTT assay. Upon reaching confluence, the cells were treated with the test materials for 24 h, after which cytokine release into the culture media was assessed using cytokine arrays RayBiotech, Inc., Norcross, GA, U.S.A. The microarrays were scanned with an Axon GenePix 4100A (Molecular Devices, LLC, Sunnyvale, CA, U.S.A.) Scanner analysed with GenePix Pro software.

### Methods: UV-irradiation clinical study (Antioxidant Peptide Serum)

To evaluate the benefits of the APS, a double-blind, single-centre clinical study was conducted to compare the efficacy of APS with two other topical products: one that contained vitamins C and E (C+E) and one that contained vitamins C and E with ferulic acid (CEF). A modification of an established pigmentation spot model was utilized 2009. The criteria for participation in the twenty-day study included male or female subjects aged 18 years or older with Fitzpatrick skin types III-IV. At baseline, the MED for each subject was determined using a solar simulator with spectral output comparable to that of natural solar radiation (UVB: 290-320 nm, UVA: 320-400 nm). As per 21 Code of Federal Regulations (CFR 50.25), written informed consent was obtained from each subject prior to enrolment into the study.

**Figure 2:**
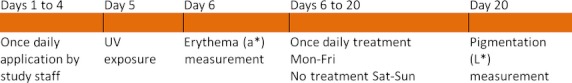
UV-irradiation clinical study treatment schedule.A schematic representation of the treatment schedule for the UV irradiation clinical study.

*Treatment Application*: Four test areas were marked onto the backs of the subjects including an untreated control, APS, C+E and CEF. The assignments of the test products to the test areas were randomized to avoid site bias. Thirty microlitres of each test product was applied to the respective sites, once daily, by the study staff as per the treatment schedule (Fig. 2).

*Analysis*: Standardized digital photographs were taken of the test sites at Day 6 and at Day 20. The images were analysed using a computer-aided colorimetry algorithm according to the CIE colour standard, to determine a* (redness/erythema) at Day 6 and L* (brightness/pigmentation) at Day 20. The Protection Factor for a* and L* was then calculated for each test site. Protection Factor is defined as the per cent change from the untreated control, for each test product and measures product effectiveness versus the untreated control.

### Methods: 12-week clinical usage study (Biometa Essential Serum)

A 12-week open-label clinical study examined the efficacy and tolerability of the combination treatment serum (Biometa Complex and APS) in subjects with mild to severe facial photodamage. The criteria for study participation included female subjects aged 30 to 60 years (Fitzpatrick skin types of I-IV) with mild to severe, fine and coarse wrinkles in the periocular area. Subjects were excluded from the study if they had used topical retinoids within 3 months of the study start, received injections of dermal fillers or botulinum toxin, had facial peels, had facial resurfacing procedures within 6 months or used other forms of anti-ageing products on the face within 30 days of study start. Subjects with known allergies to the facial product regimen were also excluded.

Subjects were instructed to apply the treatment serum on their entire facial skin, twice daily (morning and evening) for twelve weeks. In addition to the treatment serum, subjects agreed to the use of a basic skincare regimen, including a cleanser, light moisturizer and sunscreen (SPF30). Subjects also agreed not to begin the usage of any new facial products other than the provided materials for the duration of the study.

IRB approval was obtained for this open-label, single-centre study from IntegReview of Austin, Texas. The study was conducted according to ethical and regulatory principles from the International Conference on Harmonization and good clinical practices. Per 21 Code of Federal Regulations (CFR 50.25), written informed consent was obtained from each subject prior to enrolment into the study.

Clinical evaluations were conducted at baseline (Visit 1), week 4 (Visit 2), week 8 (Visit 3) and week 12 (Visit 4). The following procedures were conducted at each visit:

*Efficacy Assessments*: Fine and coarse wrinkles were clinically graded using a ten-point scale, on each subject's right and left periocular area (where 0 = none, 0.5–3.5 = mild, 4–6.5 = moderate and 7–9 = severe). Coarse wrinkles were defined as wrinkles/lines that are visible at rest and are approximately greater than 1 mm in width and/or depth. Fine wrinkles are not visible at rest and are approximately less than 1 mm in width and/or depth. Ten-point scales were also used to assess skin tactile roughness (where 0 = smooth, 9 = rough), skin tone (0 = blotchy/uneven, 9 = clear/even), skin firmness (where 0 = skin appears loose and sags, 9 = skin appears firm) and skin radiance (0 = dull/flat matte, 9 = bright/luminous/radiant). All grading assessments were performed by the same investigator at each visit to ensure grading consistency.

*Safety Assessments*: Tolerability of the treatment product was assessed at all visits by the reporting of adverse events and by objective and subjective assessments. Objective irritation (erythema, oedema and scaling) was assessed by the investigator whereas subjective irritation (burning/stinging, itching and tingling) was assessed by the subject, all using a 4-point scale (where 0 = none, 1 = mild, 2 = moderate and 3 = severe).

*Standardized Photography*: Standardized, digital images were taken of the subject's left, right and frontal facial views using raking light (standard colour).

*Self-Assessment Questionnaires*: Subjects completed a Self-Assessment Questionnaire at week 12, where they rated their facial skin condition and the treatment serum's efficacy on a four-point scale (Strongly Agree, Agree, Disagree and Strongly Disagree). Subjects rated their overall satisfaction with the treatment serum on a four-point scale (1 = Excellent, 2 = Good, 3 = Fair and 4 = Poor).

*Statistical Analysis*: Mean clinical grading scores at weeks 4, 8 and 12 were compared to mean baseline scores using a Student's paired t-test. Changes from baseline were considered significant at the *P* < 0.05 level. For the subject Self-Assessment Questionnaires, the per cent incidence of positive responders was reported for the treatment serum's efficacy and aesthetic attributes sections.

## Results

### Results: *In vitro* studies (Biometa Complex)

After treatment of human dermal fibroblasts with Biometa Complex, the gene expression profiled was analysed with a special emphasis on genes implicated in the anti-ageing cascade. Table 1 lists some of the key genes regulated by Biometa Complex. Biometa Complex stimulates a number of genes involved in the extracellular matrix regeneration. Additionally, there was a downregulation of MMP, which correlated well with an upregulation of tissue inhibitor of metalloproteinase (TIMP). Genes necessary to maintain the overall health of the skin including stress proteins such as heat shock protein (HSP), antioxidant proteins such as superoxide dismutase (SOD1) and proteins important for maintaining skin barrier such as tight junction proteins (TJ1) were upregulated.

**Table 1:** Genes regulated by Biometa Complex

Function	Genes	Brief description
Extracellular matrix proteins	Col 1A1, COL12A1, COL15A1	Genes encoding collagen
Growth factors and cytokines involved in repair and ECM production	TGF-beta1	Induces synthesis and secretion of major extracellular matrix proteins, collagen and elastin
	TIMP1/TIMP3	TIMPs play an important role in the regulation of MMP activity
	VEGFA/VEGFb	Key role in angiogenesis
	FGF3	Enhanced hyaluronan production and implications in tissue remodelling
	IGF	Enhanced hyaluronan production and implications in tissue remodelling
	IL10RB, IL11, IL8, etc	Involved in several signal transduction pathways that include tissue remodelling, etc
Antioxidant	SOD1	Involved in regulation of ROS-mediated tissue damage, usually found in the extracellular matrix and is ideally situated to prevent cell and tissue damage initiated by ROS
Stress related	HSP	Heat shock proteins are part of cellular defence against stress
Skin barrier	TJP1/ZO-1	Help improve skin barrier function

Protein analysis of medium after treatment demonstrated that Biometa Complex stimulates fibroblasts to express and synthesize key proteins known to be involved in skin repair and remodelling including fibroblast growth factor (bFGF), TIMP-1 and vascular endothelial growth factor (VEGF). A good correlation between gene expression and protein synthesis was seen. These results strongly suggest that influences on these strategic fibroblast proteins could lead to anticipated anti-ageing benefits.

### Results: UV-irradiation clinical study (Antioxidant Peptide Serum)

Seventeen healthy female volunteers between the ages of 26 and 63 years with Fitzpatrick skin types III-IV completed the twenty-day study.

**Figure 3:**
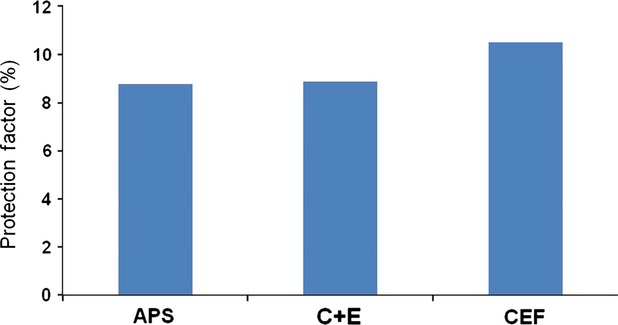
Significant protection against UV-induced erythema at Day 6. All three products provided significant protection against UV-induced erythema compared to untreated control (all *P* ≤ 0.025).

*UV-induced erythema*: At Day 6, all three test products (APS, C+E and CEF) provided statistically significant protection from UV-induced erythema compared to untreated control (all *P* ≤ 0.025), when applied once daily for four days prior to UV irradiation. The products did not perform significantly different from each other (Fig. 3).

**Figure 4:**
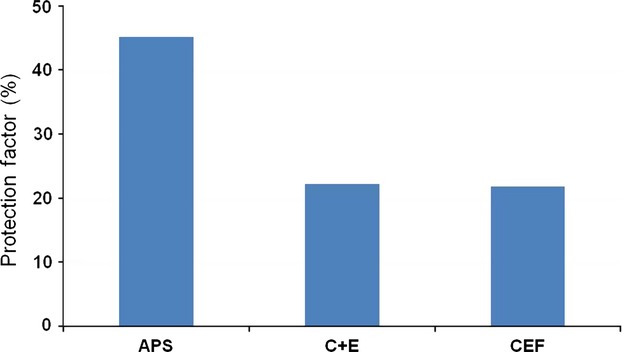
Significant protection from UV-induced pigmentation at Day 20. APS provided significantly greater protection against UV-induced pigmentation over CEF and C+E (*P* < 0.0001).

*UV-induced Pigmentation*: At Day 20, all three test products provided statistically significant protection from UV-induced pigmentation compared to untreated control (all *P* < 0.001), when applied once daily for eleven days following UV irradiation. Particularly of note, APS provided significantly greater protection over both C+E and CEF serums from UV-induced pigmentation (*P* < 0.0001) as shown in Fig. 4.

### Results: 12-week clinical usage study (Biometa Essential Serum)

Thirty-three female subjects aged 35–56 years with mild to severe, fine and coarse periocular wrinkles were enrolled and completed the twelve-week study.

**Figure 5:**
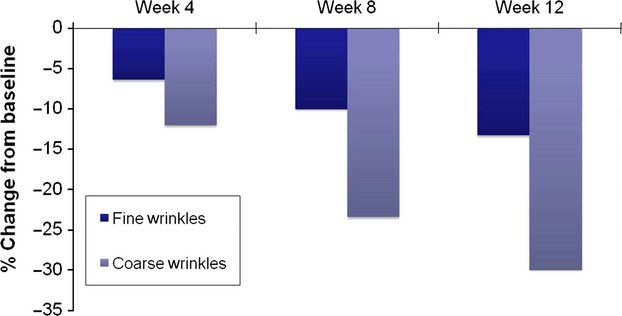
Investigator assessments of fine and coarse wrinkles. Significant reductions in mean scores for fine and coarse wrinkles at all visits (all *P* < 0.0001).

**Figure 6:**
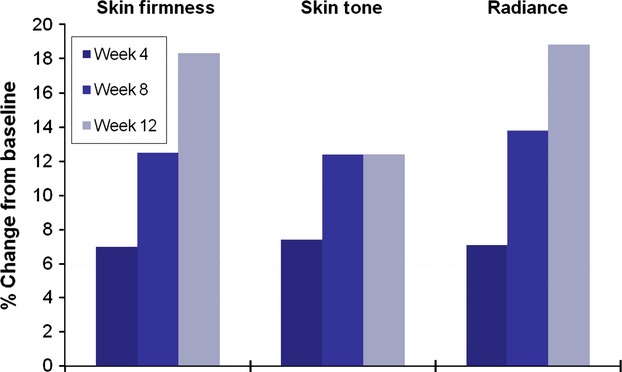
Investigator assessments of skin tone, firmness and radiance. Significant improvements in mean scores for skin firmness, skin tone and radiance at all visits (all *P* ≤ 0.02).

**Figure 7:**
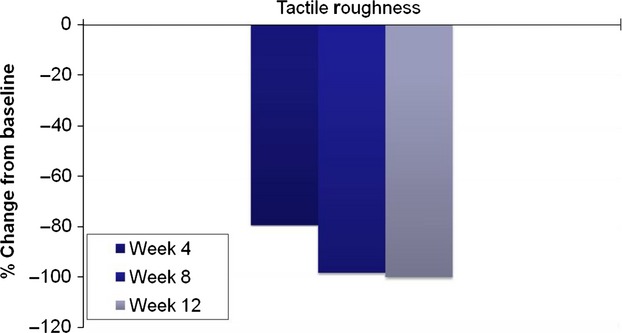
Investigator assessments of tactile roughness. Significant improvements in mean scores for skin firmness, skin tone and radiance at all visits (all *P* < 0.0001).

At weeks 4, 8 and 12, statistically significant improvements in mean scores for all photoageing parameters were achieved after twice daily topical use of the combination treatment serum. Investigator assessments demonstrated significant reductions in mean scores for fine and coarse periocular wrinkles at week 4 with continued significant reductions through week 12 (all *P* < 0.0001) as shown in Fig. 5. Coarse periocular wrinkles decreased by 30% from baseline at week 12. Skin firmness, skin tone, radiance and tactile roughness reflected similar significant and progressive improvements from baseline at all follow-up visits (all *P* ≤ 0.02) as depicted in Figs 6 and 7. Especially prominent was the improvement in the texture of the skin achieved at the first visit (week 4), as represented by a significant reduction (79.5%) in mean tactile roughness scores (*P* < 0.0001).

**Figure 8:**
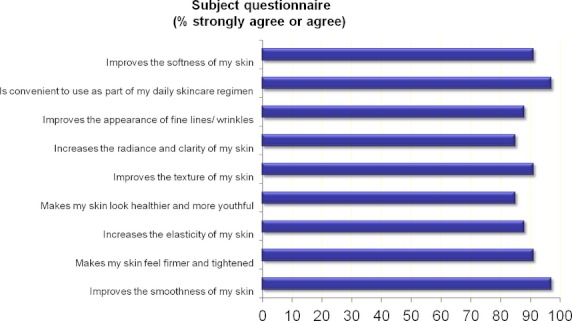
Subject self-assessment questionnaire at week 12. The Biometa Essential Serum was highly rated by subjects after 12 weeks of use as reflected in the percentage of subjects who selected Strongly Agree or Agree to the questions presented above.

The subject responses in the Self-Assessment Questionnaire at week 12 strongly support the improvements observed in the investigator assessments, with over 85% of subjects responding favourably to all questions. The percentages of subjects who selected Strongly Agree or Agree in response to the questionnaire at week 12 are presented in Fig. 8.

**Figure 9:**
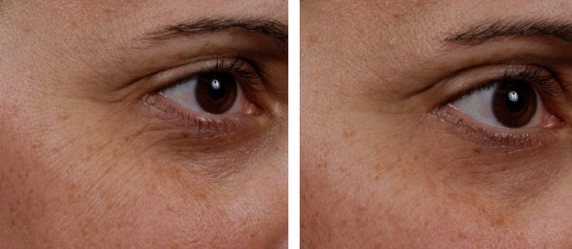
Improvements in peri-ocular fine and coarse wrinkles and skin tone were demonstrated in a 49 year old Caucasian female subject with Fitzpatrick skin type III, after four weeks of product use.

**Figure 10:**
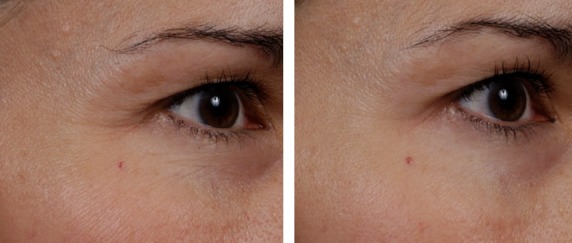
Improvements in fine peri-ocular wrinkles, skin tone, tactile roughness and radiance at week 4 in a 46 year old Hispanic female subject with Fitzpatrick skin type III.

**Figure 11:**
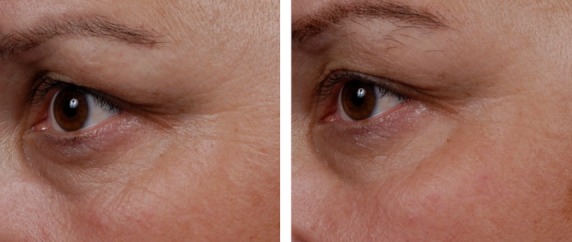
A 46 year old Korean/Caucasian female subject with Fitzpatrick skin type II, presenting with fine and coarse peri-ocular wrinkles and uneven skin tone visibly improved after four weeks of product use.

Standardized digital photographs displayed in Figs 1 represent examples of clinical responses after twice daily use of the combination treatment serum. Improvements in periocular fine and coarse wrinkles and skin tone were demonstrated in a 49-year-old Caucasian female subject with Fitzpatrick skin type III, after four weeks of product use (Fig. 9). In addition, a 46-year-old Hispanic female subject with Fitzpatrick skin type III shows visible improvements in fine periocular wrinkles, skin tone, tactile roughness and radiance at week 4 (Fig. 10). A 46-year-old Korean/Caucasian female subject with Fitzpatrick skin type II, presenting with fine and coarse periocular wrinkles and uneven skin tone, visibly improved after four weeks of product use, as displayed in Fig. 11.

The combination treatment serum was well tolerated, and there were no treatment-related adverse events reported during the course of the study. Mean scores for oedema, scaling, burning/stinging, itching and tingling remained less than mild throughout the study (all scores <0.09 on the 0–4 scale). Notably, mean scores for erythema actually decreased from baseline at all follow-up visits, with a statistically significant decrease observed at week 8 (*P* < 0.03).

## Discussion

Incorporating knowledge of biochemical mechanisms involved in skin ageing along with an increased understanding of cosmetic ingredients and their associated target activity is a rational approach to the development of next generation anti-ageing products. A combination product that addresses protection and repair as well as long-term effect on overall skin health is likely to produce the most significant results in reversing the signs of skin ageing.

Increasing evidence suggests the central role of ROS in the mechanisms of both intrinsic and extrinsic ageing 2002. At the molecular level, ROS causes a cascade of biochemical events in the skin resulting in the production of MMPs and proinflammatory cytokines. UV-induced MMP initiates cleavage of fibrillar collagen (type I and III in skin), impairing the structural integrity of the dermis. Such cumulative collagen damage is likely a major contributor to the phenotype of photodamaged human skin 2009. Additionally, UV irradiation impairs ongoing collagen synthesis by down regulating type I and III procollagen gene expression (possibly by induction of AP-1 and downregulation of TGF-beta) 2002.

This sun-induced damage to dermal collagen, elastin and ECM components coupled with impairment of their repair processes, ultimately lead to loss of structural integrity in the dermis, increasing the number and severity of wrinkles and worsening skin texture, key clinical features of aged appearance 2002.

The product described herein addresses protection from ROS and the downstream effects of ROS by boosting collagen production, decreasing collagen degradation and pigmentary changes. In addition to such protection, it is necessary to maintain the overall health of the skin and reverse the function of older skin cells to that of younger skin cells by stimulating genes involved in the anti-ageing cascade.

As discussed previously, one chamber contains Biometa Complex. Biometa Complex is a complex mixture of microbial growth factors, peptides, oligosaccharides and other important cellular components derived through a proprietary fermentation process that employs Pichia pastoris to metabolize conditioned nutrients. It is believed that the process of metabolization allows for greater bioavailability of the important ingredients to the skin. *In vitro* studies have demonstrated that this complex can stimulate fibroblasts to express a number of key genes involved in the anti-ageing cascade (Table 1). Some of the key genes that are upregulated include: (1) COL1A1 (Collagen, type I, alpha 1): human gene that encodes the major component of type I collagen, (2) TGF-beta: known to induce synthesis and secretion of major extracellular matrix proteins, collagen and elastin, (3) VEGF: plays a key role in angiogenesis, (4) FGF3: enhanced hyaluronan production and implications in tissue remodelling, (5) SOD-1: helps in regulation of ROS-mediated damage, usually found in the extracellular matrix and is ideally situated to prevent cell and tissue damage initiated by ROS, (6) HSP: protection from external stress stimuli, (7) TJP1: improve skin barrier function and (8) TIMP1/TIMP3: natural inhibitor of MMPs. Biometa Complex downregulated the expression of MMP. These data suggest that Biometa Complex is capable of rejuvenating fibroblasts.

The second chamber of the treatment serum contains a concentrated blend of ingredients including antioxidants, extracellular matrix-building peptides and skin-lightening agents. Palmitoyl tripeptide and dipalmitoyl hydroxyproline were included in the formulation for their effects on collagen structure and function. The treatment serum also contains a combination of scientifically proven antioxidants such as vitamins C and E, ergothioneine, blackberry leaf extract, coenzyme Q-10, green tea extract, sericin and saccharomyces ferment filtrate. The treatment serum also contains alpha-arbutin, which has been shown to reduce skin pigmentation by multiple mechanisms 8, 9 and hyaluronic filling spheres to provide a rapid smoothing effect on wrinkles.

The effectiveness of the combination serum's multiple ingredients in targeting key skin repair pathways as described above is reflected clinically in both the UV-induced erythema and pigmentation model study and a three-month clinical usage study. Statistically significant improvements in both fine and coarse periocular wrinkles, skin firmness, skin tone, radiance and tactile roughness were achieved after only four weeks of twice daily topical use, with continued significant improvements at all subsequent time points. Notably, a reduction in erythema was observed in both the clinical studies. Improvement in pigmentation and skin tone was also independently observed in both studies: significant reduction in UV-induced hyperpigmentation and significant improvements in uneven skin tone. Overall, the results from these clinical studies consistently reflect the combination serum's ability to improve the clinical manifestations of photodamaged or aged skin.

## Conclusion

This treatment serum provides physicians and patients with a unique and effective topical combination product to improve the signs of skin ageing. Future work should be directed at identifying new targets in the multiple pathways involved in process of skin ageing and repair and to evaluate various combinations of ingredients to optimize efficacy.
